# Accumulation of magnetite by flotation on bubbles during decompression of silicate magma

**DOI:** 10.1038/s41598-019-40376-1

**Published:** 2019-03-07

**Authors:** Jaayke L. Knipping, James D. Webster, Adam C. Simon, François Holtz

**Affiliations:** 1 Institut für Mineralogie, Leibniz Universität Hannover, Callinstraße 3, 30167 Hannover, Germany; 2 Department of Earth and Planetary Science, American Museum of Natural History, Central Park West at 79th Street, New York, NY 10024-5192 USA; 3 Department of Earth and Environmental Sciences, University of Michigan, 1100 North University Ave, Ann Arbor, Michigan 48109-1005 USA

## Abstract

Magnetite (Fe_3_O_4_) is an iron ore mineral that is globally mined especially for steel production. It is denser (5.15 g/cm^3^) than Earth’s crust (~2.7 g/cm^3^) and is expected to accumulate at the bottom of melt-rich magma reservoirs. However, recent studies revealed heterogeneous fluid bubble nucleation on oxide minerals such as magnetite during fluid degassing in volcanic systems. To test if the attachment on fluid bubbles is strong enough to efficiently float magnetite in silicate magma, decompression experiments were conducted at geologically relevant magmatic conditions with subsequent annealing to simulate re-equilibration after decompression. The results demonstrate that magnetite-bubble pairs do ascend in silicate melt, accumulating in an upper layer that grows during re-equilibration. This outcome contradicts the paradigm that magnetite must settle gravitationally in silicate melt.

## Introduction

**Figure 1:**
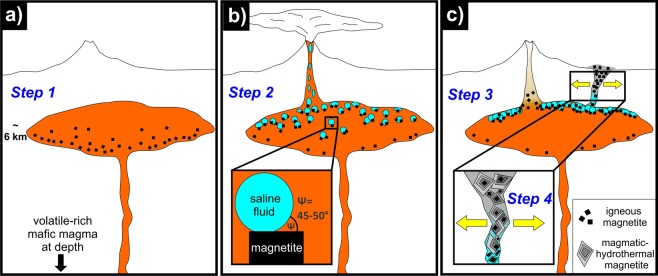
Schematic illustration of the magnetite-flotation model for Kiruna-type iron oxide-apatite deposits^10^. **(a)** Primary igneous magnetite crystallizes from silicate melt in a magma reservoir and should gravitationally settle owing to its higher density relative to melt. However, (**b**) if saline fluid exsolves during decompression and bubbles nucleate on magnetite crystals owing to favorable wetting properties, then **(c)** magnetite-bubble pairs form and buoyantly ascend, coalesce and separate as a magnetite-fluid suspension within the magma, and can escape the magma if extensional tectonic stress opens crustal fractures wherein secondary magmatic-hydrothermal magnetite can precipitate, at lower pressures and temperatures, and surround primary igneous magnetite crystals.

Fractional crystallization in transcrustal magmatic systems is a fundamental control on magma differentiation, wherein gravitational settling and flotation of minerals based on density contrasts causes compositional evolution of magmas and, in turn, the evolution of Earth’s crust^1^. Accordingly, minerals with a density less than Earth’s crust (~2.7 g/cm^3^), such as plagioclase (2.6–2.7 g/cm^3^), are separated by mineral flotation^2^, while dense ore phases such as sulfide melt droplets and oxide minerals (e.g., magnetite: 5.15 g/cm^3^, chromite: ~4.5 g/cm^3^) are separated by gravitational settling. However, flotation of dense ore phases must be re-evaluated when fluid bubbles exsolve during decompression; i.e., magma ascent^3,4^. Fluid bubbles preferably nucleate heterogeneously on existing surfaces of sulfide melt droplets and oxide minerals such as magnetite and chromite^3–8^ owing to larger wetting angles (Ψ = 45–50°) when compared to silicate minerals (Ψ = 5–25°)^7^ (Fig. 1b). Actually, more than 100 years ago the mining industry took advantage of this phenomenon and shifted mineral processing methods from classical gravity separation to more efficient froth flotation wherein dense ore minerals are wetted by pine oil and injected air bubbles. The resulting mineral-bubble pairs float upwards relative to unwetted silicate minerals that sink in the reagent solutions^9^. Despite this well-demonstrated beneficiation process, the flotation of ore minerals in magma reservoirs has rarely been considered as a natural process leading to the concentration of ore minerals. Only a few studies attempted to explain ore formation by the wetting affinity between exsolved fluids and ore phases. Examples include chromite pods in podiform chromite deposits^3^, Cu-Au-rich sulfide melts in porphyry ore deposits^4^ as well as magnetite in Kiruna-type iron oxide-apatite deposits^10^ (hereafter, referred to as IOA deposits).

Genetic models proposed to explain the formation of IOA deposits are fiercely debated. IOA deposits occur worldwide and are economically important not just because of their high concentration of Fe, but also their enrichment in rare earth elements (REE) crucial for modern technologies. Classical hypotheses invoke (magmatic-) hydrothermal^11–13^ versus purely magmatic processes such as liquid immiscibility between Si-rich melt and Fe-rich melt^14–17^. In the case of the numerous IOA deposits along the Chilean Iron Belt, none of these classical models fully explain the complex textures and chemical composition of magnetite. Thus, based on observations at the world-class Los Colorados IOA deposit (~350 Mt Fe, magnetite ≤90% modal) within the Chilean Iron Belt, a novel formation model was proposed that combines the contrasting textural/geochemical observations^10^, i.e., silicate inclusion-rich magnetite cores with an igneous signature (high Ti, V, Al, Mn) surrounded by pristine magnetite with a (magmatic-) hydrothermal signature (low Ti, V, Al, Mn), into one coherent process. In this model, primary igneous magnetite crystals are proposed to form as a liquidus phase in an oxidized, hydrous andesitic magma reservoir, which are ubiquitous in arc magma systems (Fig. 1a). Upon magma ascent and decompression, saline fluids exsolve from the silicate melt and, owing to enhanced heterogeneous nucleation of fluid bubbles on magnetite surfaces, magnetite and fluid attach to each other to form a suspension that is proposed, although not demonstrated experimentally, to have a lower average density than the surrounding melt^10^ (Fig. 1b,c). Importantly, depending on the Cl concentration of the exsolved volatile phase, the magnetite-fluid suspension will contain a significant fraction of Fe dissolved as FeCl_2_ in the fluid^18^. The solubility of FeCl_2_ in fluid decreases during cooling (600–400 °C), resulting in precipitation of secondary magmatic-hydrothermal magnetite, a process that is even more effective at large decompression rates^19^. Thus, changing tectonic stress in the late Lower Cretaceous within the Atacama Fault System - host to the Chilean Iron Belt – allowed the ascent of the magnetite-fluid suspension into shallow crustal hydraulic fractures with concomitant precipitation of magmatic-hydrothermal magnetite surrounding primary igneous magnetite (Fig. 1c). This model explains the apparently contrasting geochemistry within and among magnetite grains at Los Colorados^10^ and other Chilean IOA deposits^19–21^. However, it was unclear if the attachment force between degassing bubbles and magnetite would be strong enough to segregate magnetite from silicate melt, and how much degassing is necessary for efficient separation of magnetite.

In this study, we performed high-temperature decompression experiments to test the hypothesis that magnetite flotation in a silicate melt is physically possible, and if decompression and simultaneous volatile saturation of silicate melt can lead to the formation of a magnetite-bubble suspension that has a density low enough to separate from, and ascend within, silicate melt. We assumed that the parental mantle-derived basalts in subduction zones are water-rich and lead to the emplacement of hydrous andesitic magmas in the upper crust (3–10 km)^22^. Arc-derived andesitic magmas are generally more oxidized (NNO to NNO+ 4)^23^, more hydrous (5–7 wt% H_2_O, sometimes up to 16 wt%)^22,24^ and enriched in halogens such as Cl^25^ when compared to magmas in other geologic settings. These and other parameters (see Supplementary Material S1) influence the exact depth range for possible magnetite flotation. Since Knipping *et al*.^10^ proposed arc-magmatic conditions as prerequisite for the magnetite-flotation model, we equilibrated an andesitic melt with 6 wt% H_2_O ± 1 wt% Cl at near-liquidus, fluid-undersaturated, oxidized conditions (250 MPa ≈ 6 km, 1050 °C, ~NNO + 3). The starting melt composition (andesite P1D)^26^ crystallizes magnetite as the sole liquidus phase at these conditions. All experiments were initially equilibrated for 72 h prior to isobaric quenching or isothermal decompression wherein pressure was decreased to 150 MPa before any other phase begins to crystallize (see Supplementry Material: Fig. S1). A continuous rate of ~0.025 MPa/s was chosen, which is equal to a magma ascent rate of ~0.5 m/s. The decompression experiments were either quenched immediately after decompression (t_a_ = 0 h) or they were held at elevated temperature after decompression and annealed for different time scales (t_a_ = 3 h or 72 h) to allow the ascent of magnetite-fluid bubble assemblages. After the experiments, capsules were mounted in epoxy to maintain their spatial orientation at run conditions, and the vertical walls of the capsule were removed by double-sided polishing to allow analysis of the quenched experimental magma perpendicular to the bubble ascent direction.

## Results

**Figure 2:**
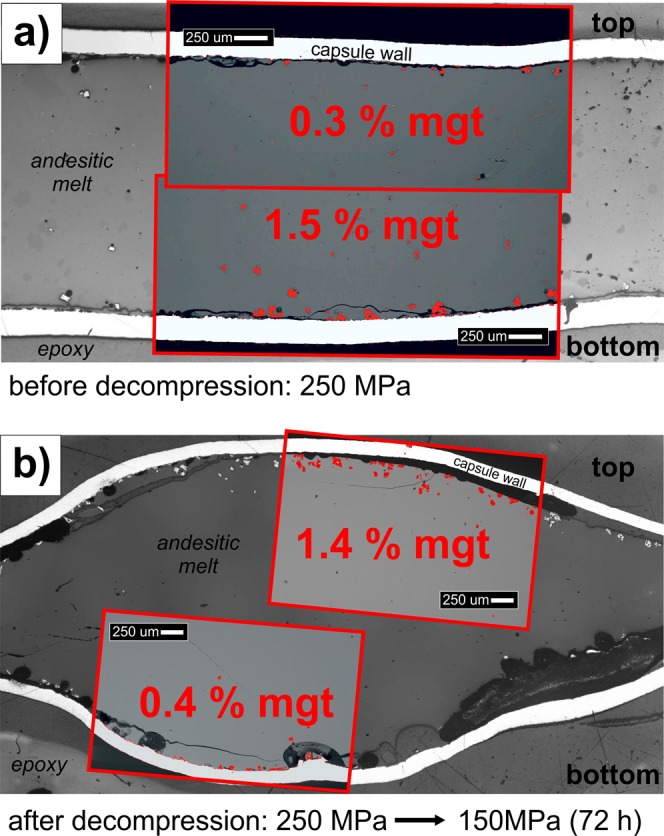
Reflected light images with backscattered-electron (BSE) image insets of H_2_O-only experiments showing andesitic glass (quenched melt), magnetite (mgt) crystals, and vesicles containing fluid bubbles. **(a)** Prior to decompression and **(b)** after decompression and 72 h annealing (t_a_ = 72 h). The phase proportion of magnetite crystals, highlighted in red, was determined quantitatively by using the software *imageJ*. Prior to decompression, the abundance of magnetite is larger at the bottom of the experimental setup (owing to gravitational crystal settling), but after decompression (and annealing) a larger concentration of magnetite is observed in the upper part of the capsule (due to magnetite-bubble ascent).

**Figure 3:**
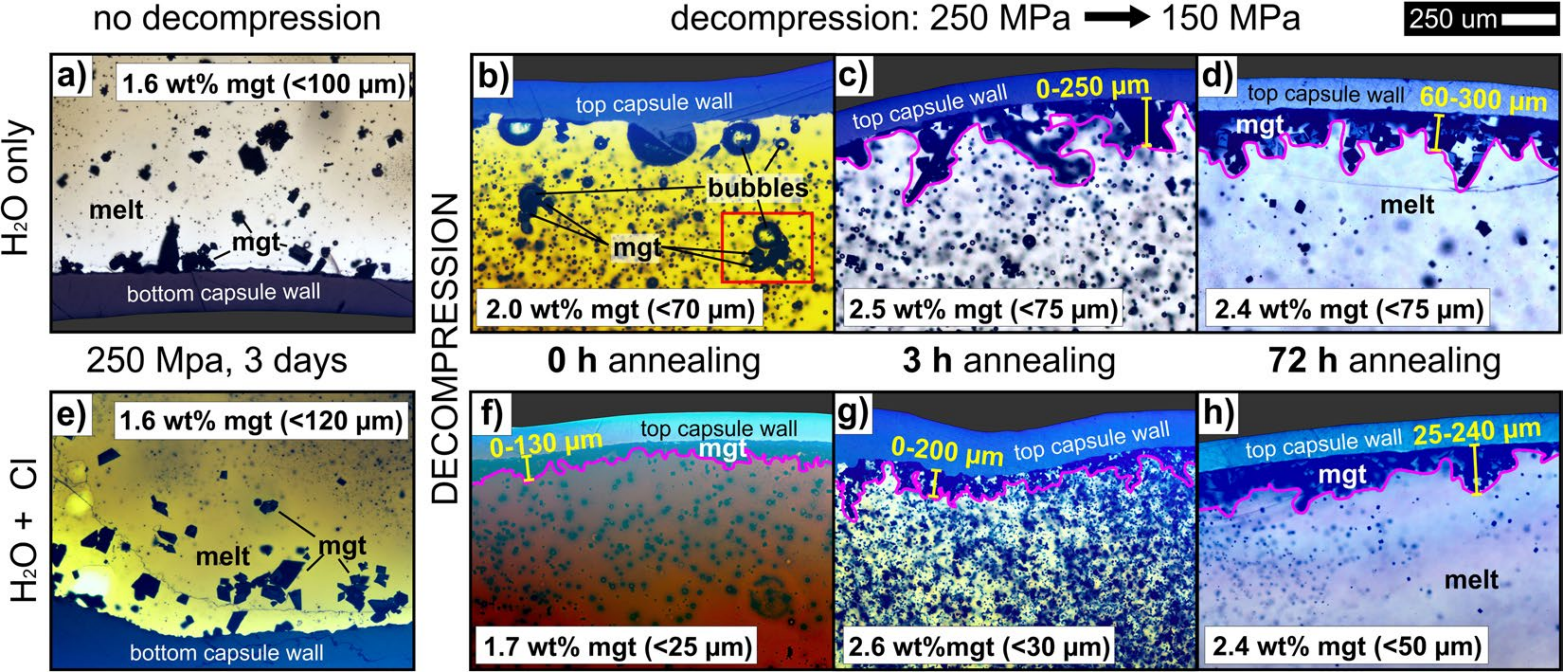
Transmitted light images of andesitic glass, magnetite crystals, and vesicles of all experiments. **(a)** to **(d)** represent H_2_O-only and **(e)** to **(h)** H_2_O + Cl experiments. **(a,e)** show the gravitational settling of large magnetite crystals at the bottom of the capsules prior to decompression (250 MPa). **(b,f)** reveal the beginning of magnetite-bubble ascent and first upper accumulation of magnetite immediately after decompression (250 → 150 Mpa, t_a_ = 0 h). **(c,g)** show the upper accumulation after t_a_ = 3 h and **(d,h)** after t_a_ = 72 h implying a growth of up to 300 μm of the upper magnetite-rich layer with increasing t_a_.

**Figure 4:**
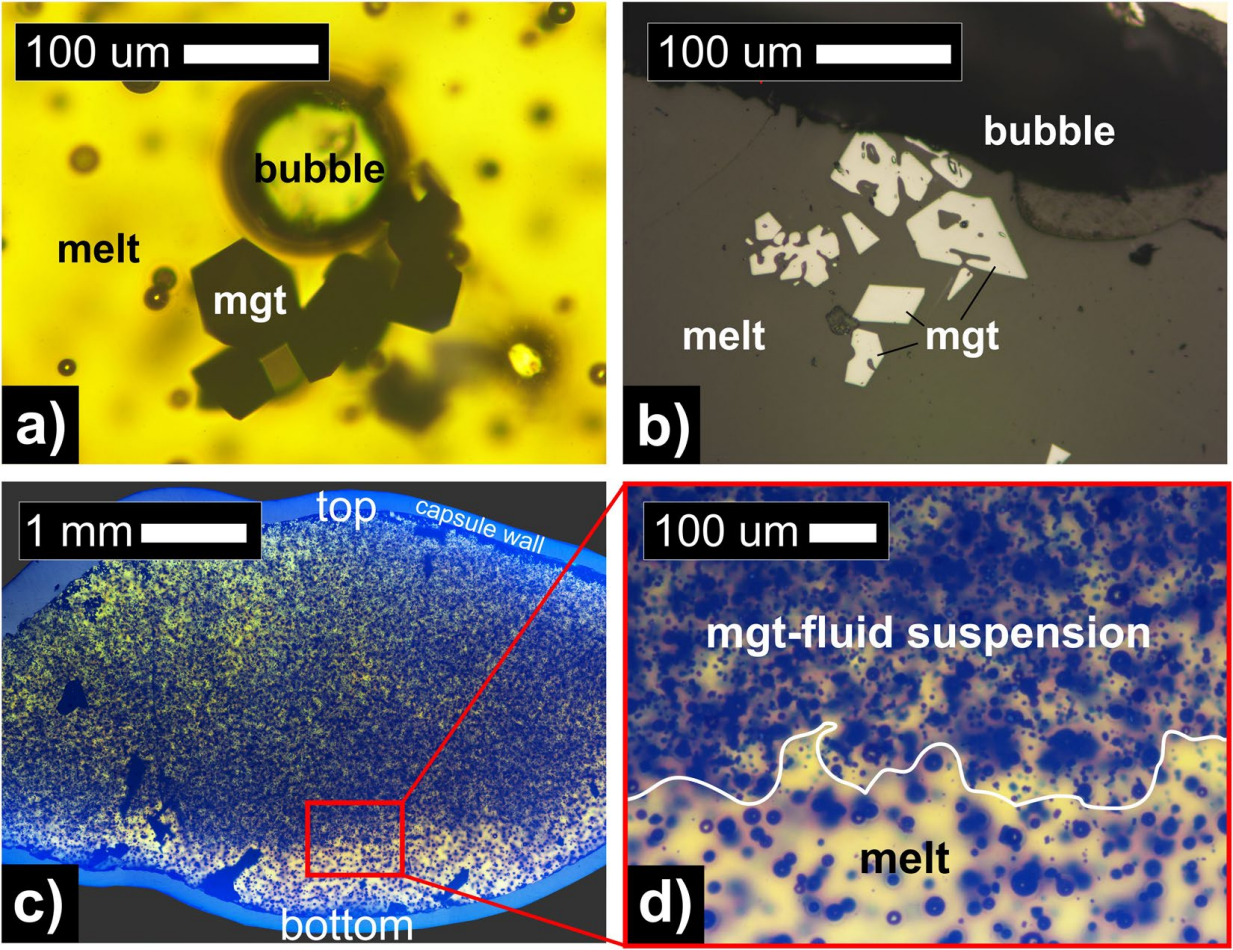
Microscopy images of andesitic glass, magnetite crystals, and vesicles in decompression experiments. **(a)** represents the inset (red rectangular) in Fig. 3b showing the microscopic process of magnetite flotation due to preferential attachment of magnetite onto an upward ascending exsolved fluid bubble (additional images in the Supplementary Material: Fig. S5). **(b)** is a reflected light image of the H_2_O-only experiment after t_a_ = 72 h exhibiting rapid magnetite (white) growth from dendritic into euhedral crystals (hopper growth) entrapping several melt (gray) inclusions. **(c)** and **(d)** are transmitted light images from the H_2_O + Cl experiment after t_a_ = 3 h that reveal the macroscopic ascent and buoyant separation of a magnetite–fluid bubble-suspension from the residual melt after decompression (see also BSE images in Supplementary Material S3: Fig. S4).

Image analysis of the isobaric, fluid-undersaturated runs (i.e., without decompression) reveals accumulation of magnetite crystals that settled to the bottom of the melt for both the H_2_O-bearing (Fig. 2a) and the H_2_O + Cl-bearing experiments. The measured thermal gradient across the charge was always ≤5 °C; thus, gravitational force is the only explanation for spatial heterogeneity of magnetite crystals. However, after decompression and subsequent annealing, magnetite accumulated efficiently in the upper part of the melt for both fluid compositions (Fig. 2b), and images of almost all decompression experiments reveal a magnetite layer at the top of the melt that becomes thicker and denser with increasing annealing time (Fig. 3). The only distinct difference caused by the fluid compositions is magnetite crystal size, which is always smaller in H_2_O + Cl-bearing decompression experiments. A smaller crystal size allows faster ascent^7^, and thus, greater upward accumulation of magnetite crystals occurs immediately after decompression to form a magnetite layer up to 130 μm thick in the H_2_O + Cl-bearing run (Fig. 3f). In contrast, larger magnetite crystals in the H_2_O-bearing experiments appear to have ascended more slowly (Figs 3b and 4a). The magnetite size limit for a positive buoyancy of bubble-magnetite pairs held together by surfaces forces ranges between 500–1000 µm^7^. Therefore, even the large crystals of the H_2_O-only bearing experiments (~75 µm) are comfortably within the range of possible flotation, as long as similar sized bubbles are present. Such large bubbles are easily produced by diffusive coarsening; i.e., Ostwald ripening within days to months^27^.

At t_a_ = 3 h, for both fluid compositions, magnetite layers of ~200 μm thickness form (Fig. 3c,g) and grow to ~300 μm at t_a_ = 72 h (Fig. 3d,h). Owing to the smaller crystal size in the H_2_O + Cl-bearing experiments, the upper magnetite-enriched layers appear to be less thick, but more dense. Larger magnetite crystals in the H_2_O-bearing experiments clearly indicate the formation of individual ascending magnetite-fluid bubble pairs in silicate magma (Figs 3b and 4a). The abundance of smaller magnetite crystals in the H_2_O + Cl-bearing experiments reveals that a large-scale process by which innumerable magnetite-fluid bubble pairs separate buoyantly as a magnetite-fluid suspension within silicate melt may be realistic (Fig. 4c,d). At t_a_ = 72 h, all exsolved fluid bubbles accumulated into a single mass located between the upper capsule wall and the the top of the melt column. Thus, no further growth of the upper magnetite layer is expected at t_a_ > 72 h and a minimum velocity of 42 µm/h of the floating suspension is estimated. Therefore, a magnetite layer of ~30 m is theoretically able to accumulate through flotation after ~2700 years on a magma reservoir scale of 1000 m (see Supplementary Material S2). The decompression experiments agree with a static run at 150 MPa, where small magnetite crystals accumulated along with exsolved fluid bubbles at the top of the melt column, in contrast to the static fluid-absent experiment at 250 MPa (see Supplementary Material: Fig. S6).

The crystal textures of magnetite also provide information for the magmatic processes involved. We observed the ubiquitous growth of magnetite crystals that appear as dendritic magnetite transitioning into euhedral crystals; i.e., hopper crystals (Fig. 4b).

## Discussion

Supersaturation caused by fast decompression rates lead to rapid, diffusion-limited crystal growth, such as hopper crystals^28^, which entrap melt inclusions within eventual large (up to ~100 μm), euhedral crystals^25^. Such melt inclusions are consistent with polycrystalline silicate inclusions observed in magnetite “cores” from IOA deposits and in chromite from podiform chromite deposits that are interpreted as igneous artifacts^10,19,21,29,30^. Our experimental results provide clear evidence that polycrystalline silicate inclusions in oxides can be primary igneous features resulting from rapid oxide crystallization from silicate melt. Abundant diffusion-limited grown dendritic magnetite grains are also observed in feeder dikes of the enigmatic El Laco IOA deposit as well as in the roof-zone of the Skaergaard layered intrusion^31,32^. In both distinctly different localities, the magnetite habit was interpreted to result from degassing-induced supersaturation^31,32^, which is consistent with our experimental results.

In the case of IOA deposits, tectonic stress changes in arc/back-arc settings cause either crustal scale venting fractures (Chilean Iron Belt; Kiruna and Grängesberg, Sweden) or caldera collapses (El Laco, Chile; St. Francois Mountains, Missouri, USA), where the opened fractures would have filled with the magnetite-fluid-suspension to form massive magnetite deposits with both igneous and hydrothermal features (Fig. 1c)^21^. In contrast, undisturbed magnetite layers are found in economically important Fe-, Ti-, V-, Cr-, and platinum group element- (PGE) mineralized layered mafic intrusions. These are intact, ancient, sill-like magma reservoirs that did not experience significant tectonic disturbance during their evolution. Layered intrusions such as the Bushveld complex and Skaergaard contain ubiquitous oxide monomineralic layers of magnetite (5.15 g/cm^3^), ilmenite (4.7 g/cm^3^) and/or chromite (4.5 g/cm^3^) that sometimes overlie less dense cumulates of plagioclase (2.6–2.7 g/cm^3^) and thus cannot be explained by typical gravitational settling^33^. Our experiments demonstrate that already a moderate amount of fluid exsolution (≤0.90 wt% H_2_O, Table S2) is sufficient for oxide flotation. Thus, even if only minor vapor saturation occurs in the melt-rich magma that overlays the crystal pile in layered intrusions, possibly enriched in H_2_O by dehydration of underlying country rocks^34^, mineral-bubble flotation should be considered a plausible process, possibly acting jointly with others, to form monomineralic oxide layers in mafic layered intrusions.

## Methods

### Experiments

All experiments were conducted in an internally heated pressure vessel (IHPV) at the American Museum of Natural History (AMNH). For this, powdered synthetic glass representative of the andesite P1D composition^26^ was loaded with 5.75 ± 0.01 wt% doubly distilled water ±1.02 wt% Cl as FeCl_3_ solution into AuPd capsules (3 mm or 5 mm in diameter) and compacted by using a piston. The capsules were welded shut and tested for leakage at T > 100 °C prior to experiments. Each experiment was loaded with two capsules, one water-only and one water + Cl-bearing capsule. All experiments were equilibrated for three days at slightly subliquidus (magnetite-bearing) water-undersaturated conditions of 1050 °C and 250 MPa and intrinsic redox conditions that are approximately NNO + 3^35^. The temperature of the charge was constantly monitored by using two K-type thermocouples at different positions of the capsule (upper left and lower right) and the measured thermal gradient was always <5 °C. Therefore, the heterogeneous spatial distribution of crystals cannot be explained by a thermal gradient. One experiment (*09-H*_2_*O* and *09-Cl*) was run at constant pressure and quenched after equilibration without decompression, while all others were decompressed isothermally after equilibration with a continuous decompression rate of ~0.025 MPa/s down to 150 MPa, which is equal to magma ascent rate of ~0.5 m/s. At this rate, water diffusion into bubbles is fast enough to maintain melt-fluid equilibrium^36^. These experiments were either quenched immediately after reaching final pressure (t_a_ = 0 h: *16-H*_2_*O* and *14-Cl*) or annealed after decompression for different durations: t_a_ = 3 h (2*8-H*_2_*O* and *2*8*-Cl*) and t_a_ = 72 h (*01-H*_*2*_*O* and *01-Cl*). After quenching, the capsules were carefully extracted from the vessel and mounted in epoxy while maintaining their original experimental orientation (top vs. bottom). In order to allow analyses perpendicular to the apparent bubble ascent direction, all capsules were mounted in epoxy, polished on both sides, and prepared as a thick section through the middle of the capsule body. For a first estimate of the magnetite distribution, reflected and transmitted light microscopy were conducted on each sample and 40–60 5x-magnified pictures were stitched together by using the software *Microsoft ICE* (e.g., Fig. 3c).

### BSE image analysis

To quantify magnetite distribution within the capsules, backscattered-electron (BSE) images were taken of the top and the bottom of each capsule using a *ZEISS EVO60 VP SEM* at the AMNH. The contrast of the images was adjusted to allow easy discrimination of magnetite from glass, capsule material and epoxy. The BSE images were afterwards analyzed by using the image analysis software *imageJ* that allowed the calculation of the phase proportion of magnetite within the glass (excluding the capsule material and epoxy). The quantification of each top and bottom area is visualized in Fig. S3 (Supplementary Material S3).

### Electron probe microanalysis

All experimental glasses were analyzed quantitatively by using a *Cameca SX-100* electron microprobe at the AMNH. Fifteen data points were collected per sample to measure the concentration of all major and minor elements other than H_2_O (Na, K, Mg, Ca, Al, Si, Ti, Fe and Cl) in the glass. An accelerating voltage of 15 kV was applied using a 10-μm beam size, beam currents of 5 nA (Na, K), 10 nA (Mg, Ca, Al, Si, Ti) and 40 nA (Cl) and counting times of 5 s (Na), 10 s (K), 20 s (Mg, Ca, Al, Si, Ti) and 120 s (Cl). Prior to each analytical session, the microprobe was calibrated by using the standards diopside (Si, Ca, Mg), jadeite (Na), orthoclase (K and Al), rutile (Ti), fayalite (Fe) and scapolite (Cl). The standardization process was checked by measuring three internal standards (basalt, andesite and rhyolite) prior and after each session. The results of the experimental glass analyses were normalized to 100% and are listed in Table S1 (Supplementary Material). Since magnetite was the only mineral phase in all samples, and Fe loss to the AuPd capsule is negligible at wet and oxidizing conditions^37^, the wt% concentration of magnetite (Fe_3_O_4_) was easily calculated from the FeO concentration in the residual glass by difference to the fully glassy starting composition P1D.

### IR-spectroscopy

In order to measure the water concentration and distribution within the samples, IR-profiles were measured perpendicular to bubble ascent direction (bottom to top) by using a *Nicolet Nexus 670 Fourier Transform Infra Red (FTIR) spectrometry system* with an attached IR Plan microscope (micro-FTIR system) at the AMNH. The spectral resolution was set to 4 cm^−1^ and five measurements were taken per sample using 200 scans. The background was analyzed after each sample. The Lambert-Beer law was applied to calculate the concentration of dissolved OH- (4500 cm^−1^) and molecular H_2_O (5200 cm^−1^) in the glass. Therefore, doubly polished glass chips (~100 μm) were prepared for the analyses and measured exactly using a micrometer (88–100 μm). The density of the glasses was estimated using the known glass composition in a density calculation model^38^. The absorption coefficients 1.27 ± 0.07 L/mol cm for molecular water and 0.84 ± 0.07 L/mol cm for hydroxyl groups in andesitic melt composition were applied^39^. The results for the total water concentrations are listed in Table S2 (Supplementary Material). Water distribution is homogeneous and no systematic variation was detected in either direction for the samples.

## Supplementary information

Supplementary Material

## Data Availability

All data is available in the main text or the Supplementary Materials.
