# Supplementary material for: Accumulation of magnetite by flotation on bubbles during decompression of silicate magma
Source: Sci Rep. 2019 Mar 7;9:3852. doi: 10.1038/s41598-019-40376-1 (PMC6405838; doi:10.1038/s41598-019-40376-1)
Supplement: Supplementary file 1 — Supplementary Material [file 41598_2019_40376_MOESM1_ESM.pdf]

Supplementary Material

Accumulation of magnetite by flotation on bubbles during decompression of silicate magma

Jaayke L. Knipping, James D. Webster, Adam C. Simon and François Holtz

S1: Pressure and temperature range of magnetite flotation

Flotation can happen from the moment of magnetite crystallization and first bubble nucleation; i.e., from the time of fluid exsolution until the density of the magnetite-bubble solution becomes higher than the surrounding melt or until the suspension reaches the top of the melt-rich magma chamber, likely a more crystalline or mushy layer. The depth range is variable and dependent on many parameters:

- water content: The higher the water content, the earlier (deeper) the fluid exsolution begins.

- density of fluid: The densiy of the fluid is dependent on the amount of dissolved $NaCl_{eq}$ and dissolved metals, such as Fe. The lower the content of solutes, the further the suspension can ascend.

- density of the surrounding melt: The density of the surrounding melt is dependent on the melt composition and dissolved water concentration. The more mafic and dryer the melt, the further the suspension can travel. However, less water content means higher viscosity of the melt and may hinder the process.

- amount of magnetite in suspension: The higher the amount of magnetite crystals in the suspension, the more difficult it is for the exsolved fluid bubbles to lift the magnetite. For example, when the abundance of magnetite exceeds 37 vol% of a suspension that contains 35 wt% $NaCl_{eq}$ and 7.2 wt% dissolved Fe in an andesitic melt with a density of 2.27 g/cm^3 (see Knipping et al. 2015a for calculation), the suspension would become negatively buoyant.

- location of the melt-rich magma reservoir in the crust: The more shallow the melt-rich magma reservoir is located (thinned crust), the shallower the suspension can ascend.

In Fig. S1, the pressure and temperature range over which magnetite flotation is possible was calculated by using the MELTS software for thermodynamic modeling for an andesite that contains 5.75 wt% H_2O at a fO_2=NNO+3. The model results indicate that magnetite flotation is possible in magma reservoirs in Earth's upper crust from ~2 to ~10 km assuming a lithostatic geobaric gradient of 28 MPa/km.

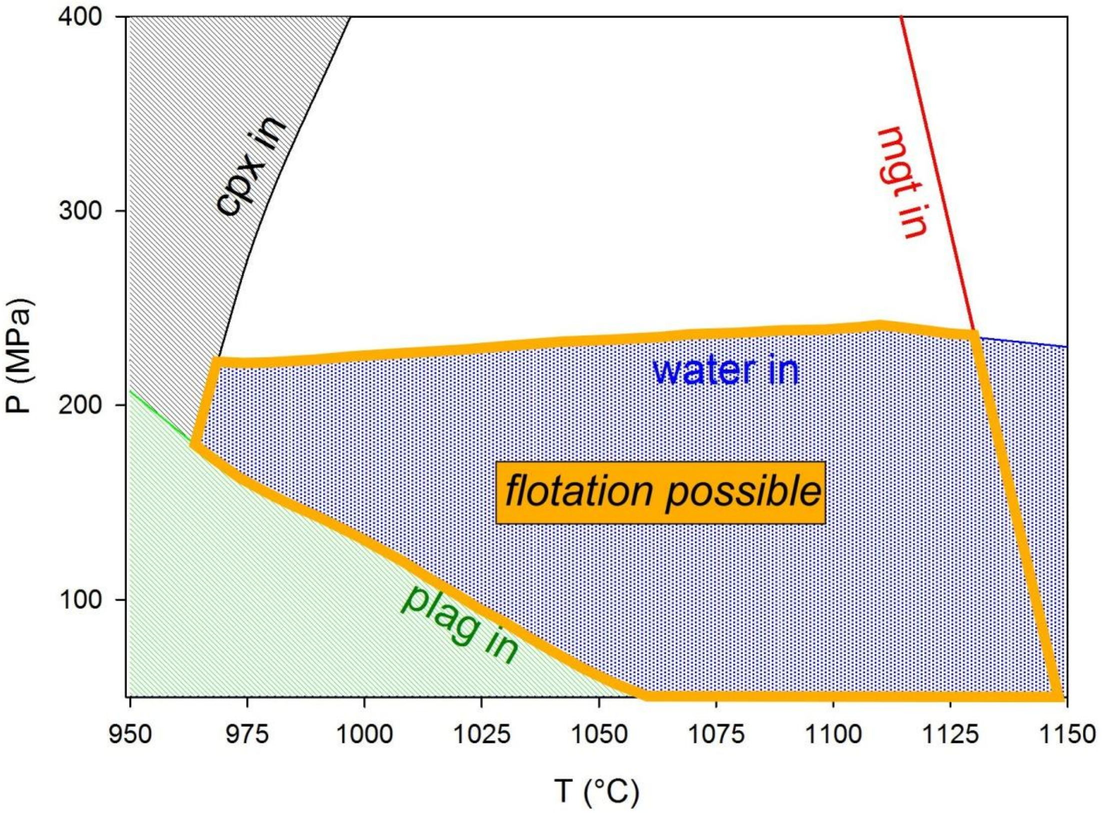

Fig. S1.
Temperature vs. pressure phase diagram calculated by using MELTS[40,41] for the P1D andesitic melt composition of Martel et al. (1999), a water concentration of 5.75 wt% H_2O, and fO_2 = NNO+3. The model results indicate that flotation of magnetite is possible over a wide range of pressures and temperatures equal to a depth range roughly from ~2 to 10 km depending on temperature.

S2: Velocity of magnetite suspension

The velocity of the magnetite suspension can be calculated by using Stoke's law (Eq. S1) due to its dependency on density contrasts, melt viscosity and bubble size.

$$v = \frac{2}{9} \times \frac{(\rho_s - \rho_{m})}{\eta} \times g \times R^2$$

Eq. S1

ρ_s equals the density of the magnetite-fluid-suspension, which is dependent on the proportion of magnetite (5.2 g/cm^3) and fluid (0.5 g/cm^3) in the suspension, ρ_m equals the melt density (2.27 g/cm^3)[10], η equals melt viscosity (2.1 log kg/m*s)[42], g is the gravitational force (9.81 m/s^2) and R is the bubble radius. Stoke's law usually calculates the sinking velocity of particles. Therefore, positive buoyant particles have a negative velocity. Fig. S2a shows the velocity range for different magnetite and bubble sizes on an experimental scale, while Fig. S2b displays the velocity on a more natural scale; i.e., m per 1000 years. Smaller magnetite grains require smaller bubbles in order to reach the same velocity. In general, magnetite and bubble sizes overlap the observations from the experiments. The results indicate that magnetite grains measuring 10 and 20 µm can be easily lifted by bubbles that are >15 or >32 µm diameter, respectively. Larger magnetite crystals of 50 µm require larger bubbles of > 82 µm.

According to the experiments, re-equilibrium is reached after at least 72 h; i.e., by 72 h all bubbles ascended through the melt column and accumulated between the capsule wall and the melt and no bubbles are anymore existent within the melt. Thus, a minimum velocity of 3000 µm/72 h = 42 µm/h can be assumed for the suspension. This velocity translates to at least 365 m/1000 years on a natural scale. In a 1000 m thick magma reservoir it would take approximately (1000 m / 0.365 m/a =) 2700 years to reach re-equilibrium; i.e., to theoretically float all bubble-magnetite-pairs that could ideally accumulate into a (100 µm/3000 µm * 1000 m =) 33 m thick magnetite layer at the roof. Therefore, magnetite flotation is a very fast and efficient process on a geologic scale.

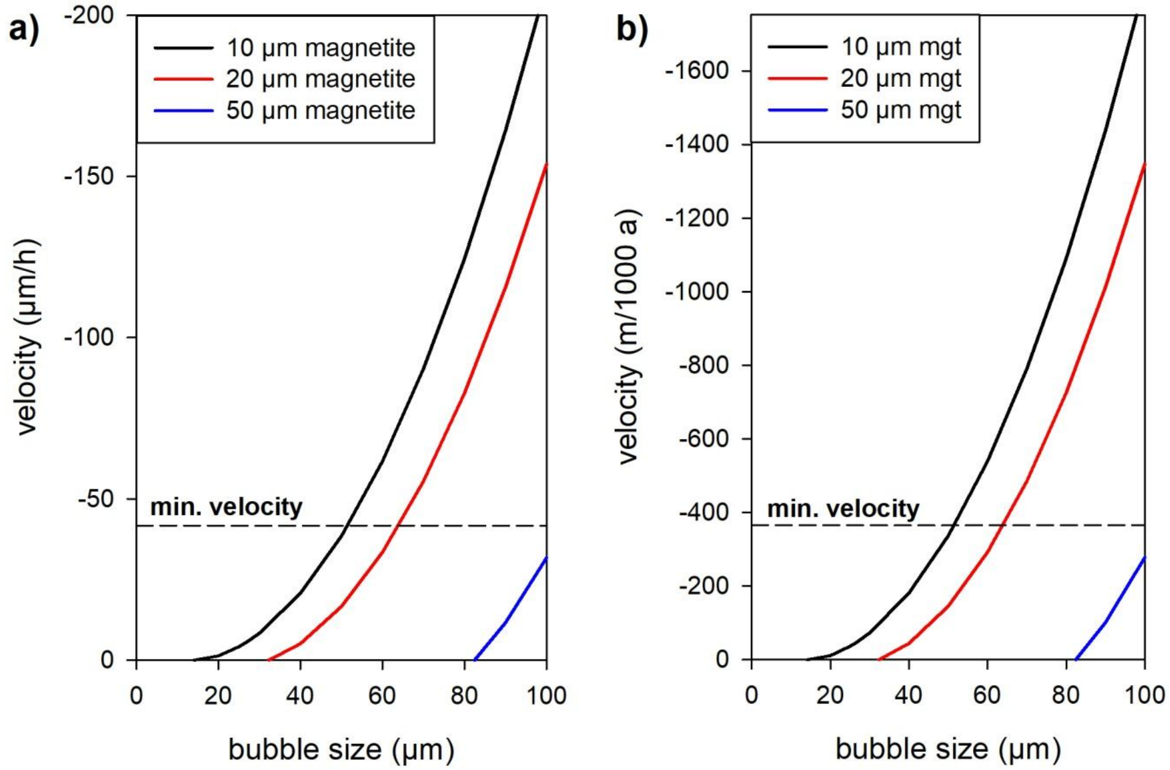

Fig. S2.
Bubble size vs. velocity of suspension shown for different magnetite sizes. **a)** shows the velocity on an experimental scale (μm/h), and **b)** shows the velocity on a geologic scale (m/1000 a). The horizontal line implies the minimum velocity estimated from the experiments.

S3: Overview of all experiments

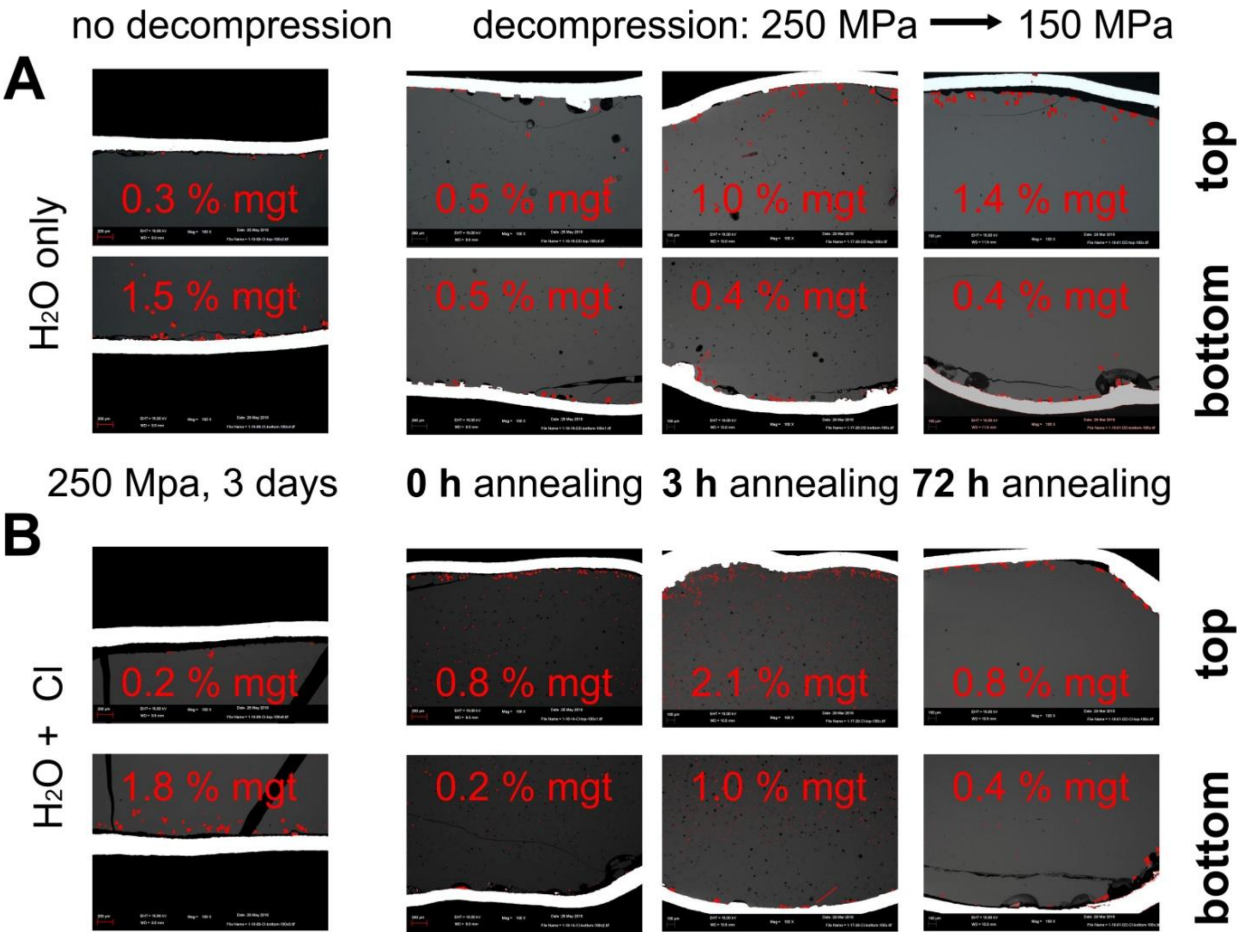

Fig. S3.

BSE images of top and bottom areas of andesitic glass, vesicles and magnetite in all experimental capsules. Panel **(A)** shows the H_2O-only runs and panel **(B)** shows the H_2O+Cl runs. Magnetite (mgt) is highlighted in red and was proportionally counted in the glass (gray) by using the software *imageJ*. For the area calculations, the outer areas such as capsule material (white) and epoxy (black) were excluded from total area. *Noteworthy:* The indicated percentage areas of magnetite cannot be equalized to the actual weight percentage of magnetite present, but they do represent the shifting ratios from the bottom of the melt column to the top of the melt column after decompression and annealing.

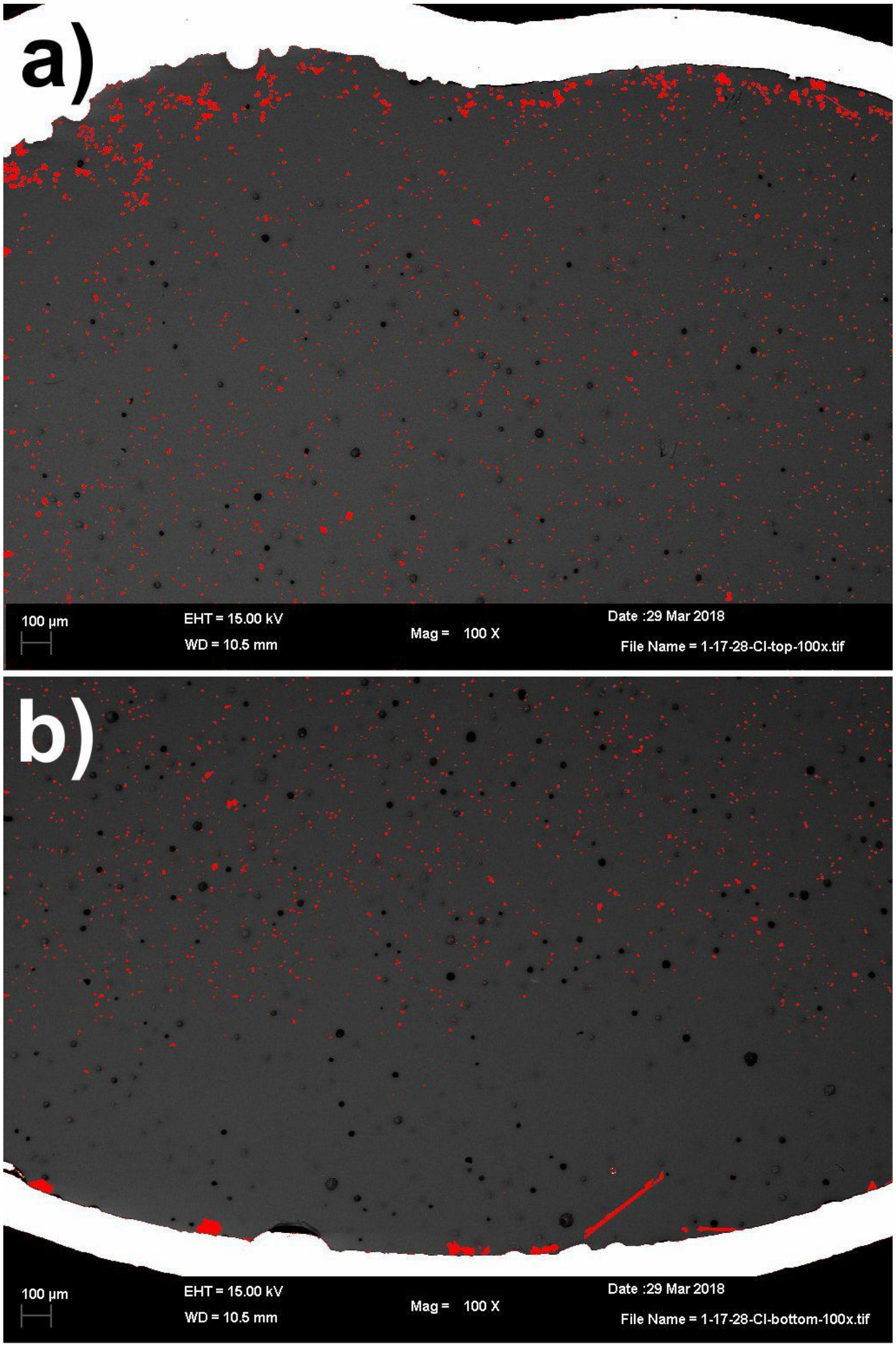

Fig. S4.
Enlarged BSE images from top **(a)** and bottom **(b)** of the Cl-bearing experiment (Fig. S3B) equilibrated for 3 h. Magnetite is highlighted in red, bubbles are black, capsule wall is white and andesitic glass is grey. After 3 h magnetite has clearly accumulated at the top of the melt column and is depleted in the bottom of the melt column aside from a few large magnetite crystals that settled to the bottom of the melt column.

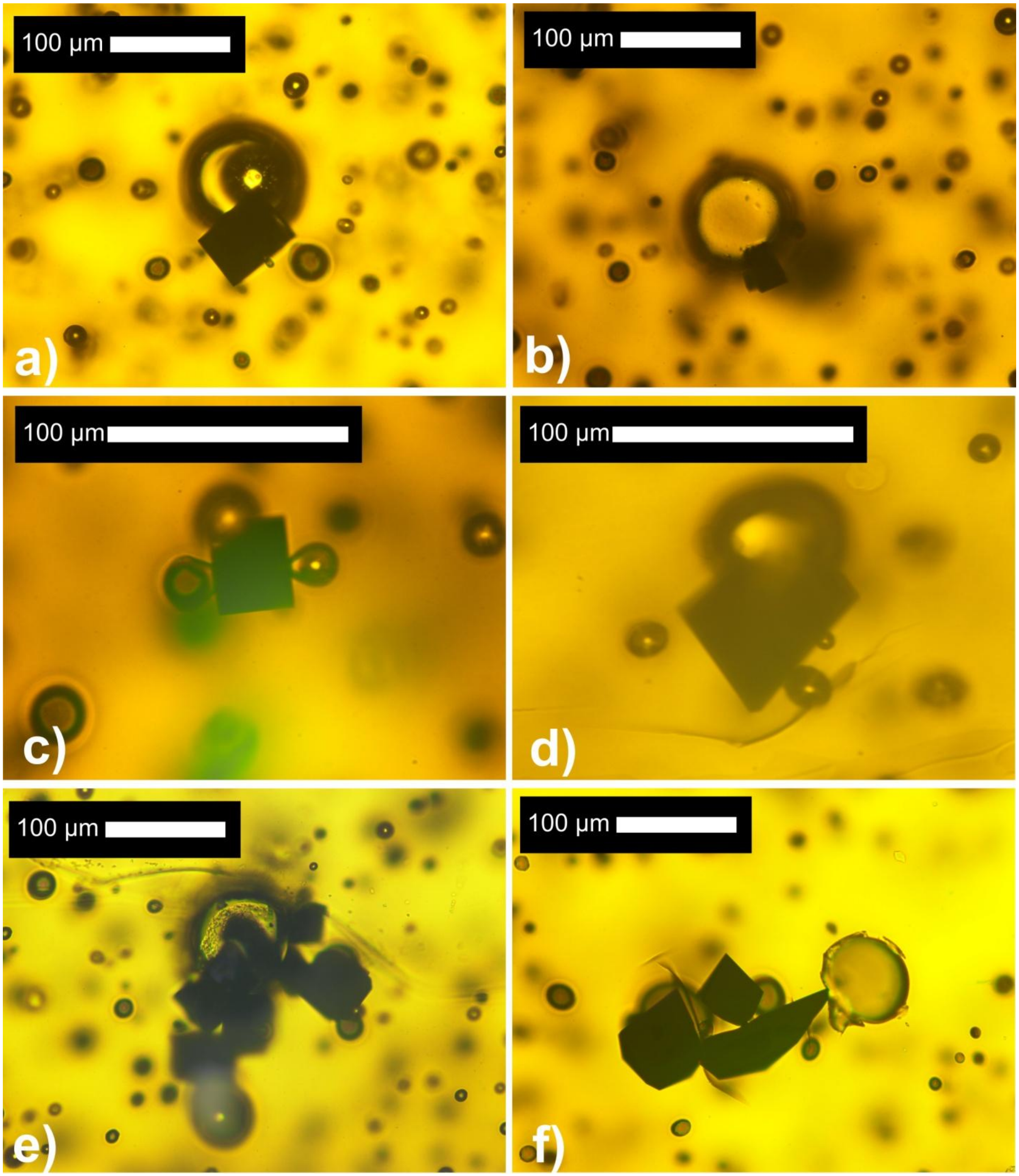

Fig. S5.
Zoomed-in transmitted light images of the decompressed H_2O-only run directly quenched after decompression (in addition to Fig. 4a). Magnetite is either wetted by one bubble **(a,b)**, by several bubbles **(c,d)** or magnetite aggregates are attached to one or more bubbles **(e,f)**.

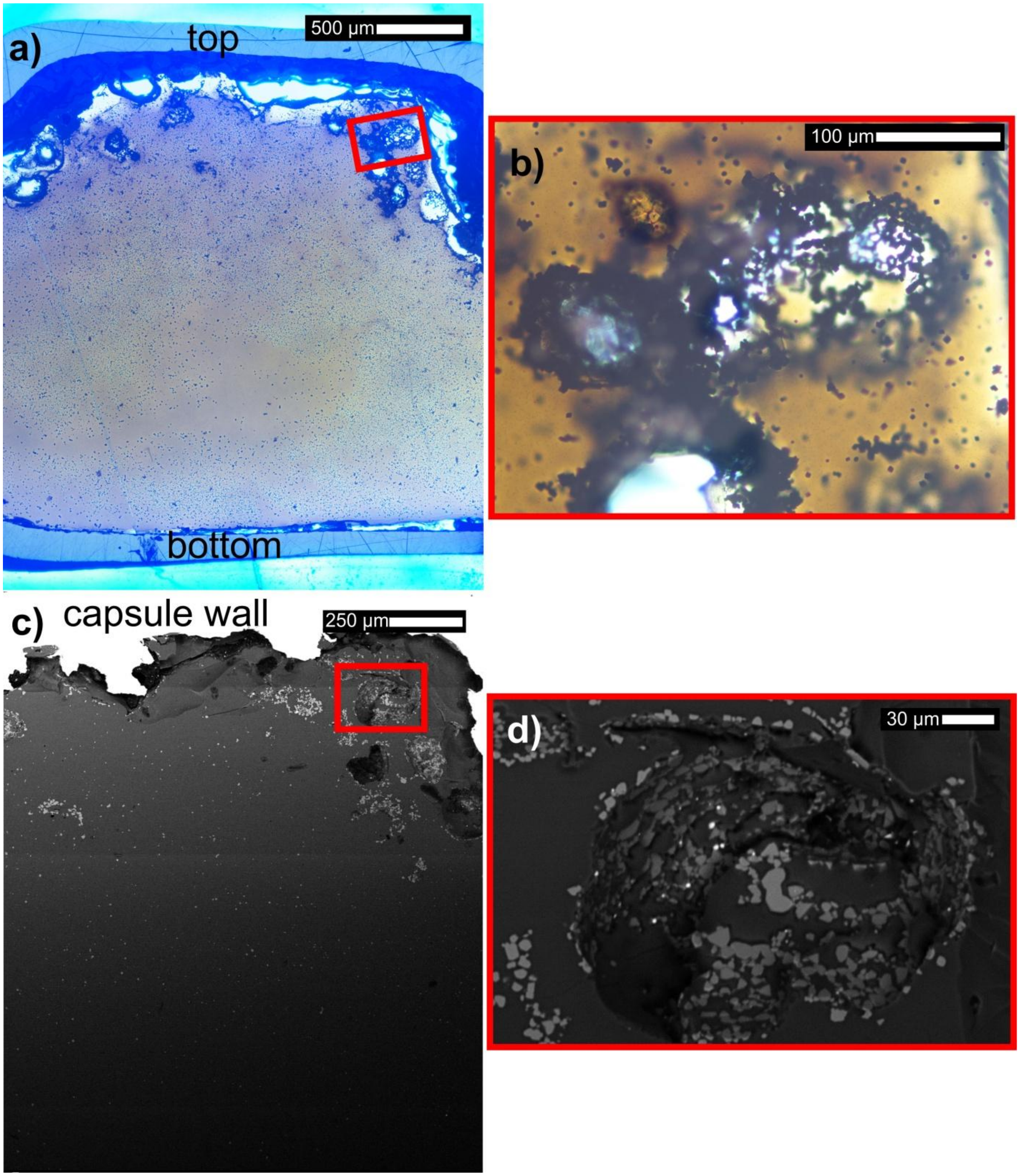

Fig. S6.
Transmitted light **(a,b)** and BSE **(c,d)** images of an experiment conducted at a constant final pressure of 150 MPa without prior decompression and equilibrated for 3 days. The results reveal a heterogeneous distribution of magnetite and exsolved fluid bubbles. Innumerable small magnetite crystals (< 10 µm) are efficiently attached to the exsolved fluid bubbles that accumulated at the top of the capsule, while the bottom of the capsule is depleted in magnetite. This is in contrast to the fluid-absent static experiment at 250 MPa wherein large magnetite crystals (< 100µm) settled gravitationally to the bottom of the melt column (Fig. 3a,e).

S4: Crytal habits

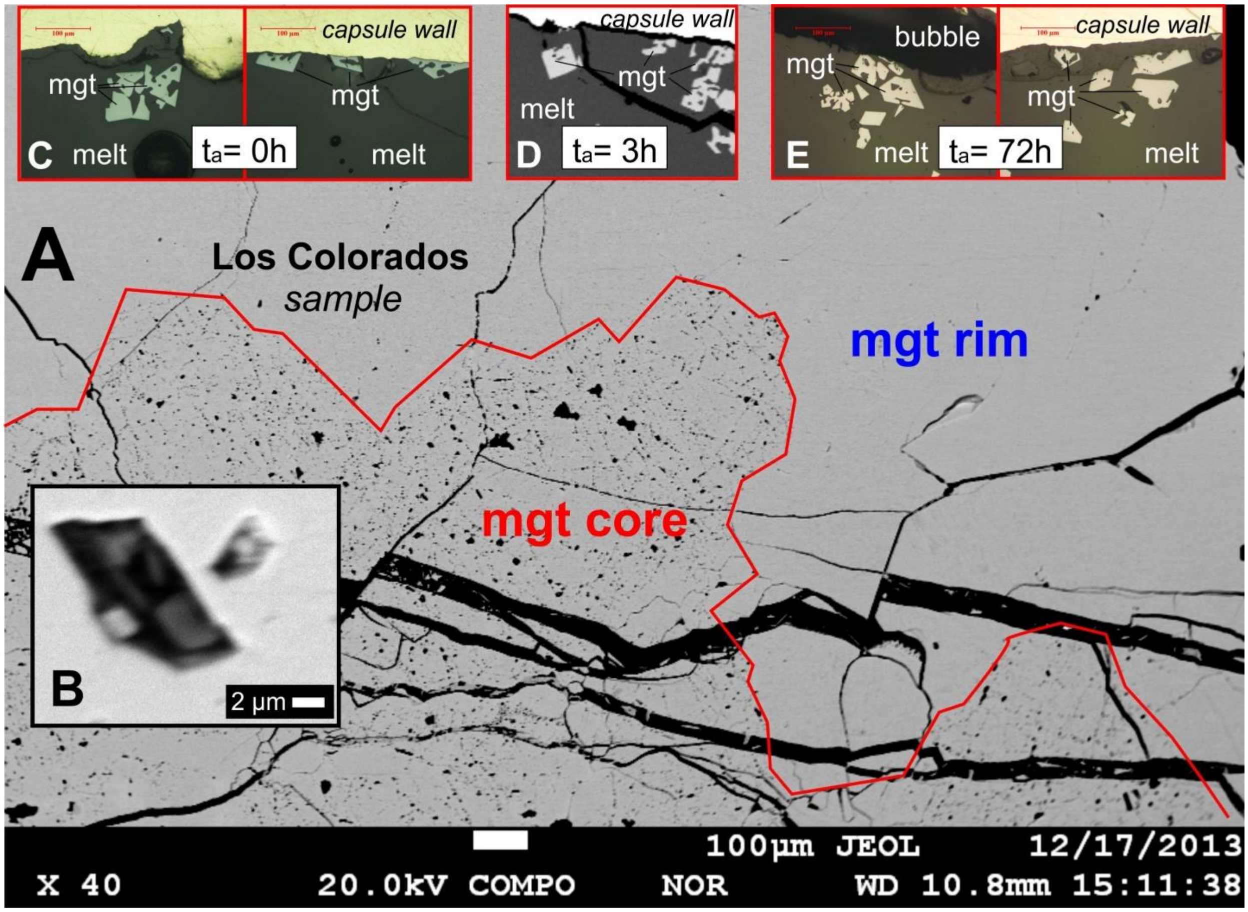

Fig. S7.

BSE image of a natural magnetite sample from the Los Colorados IOA deposit in comparison with reflected light and/or BSE images of magnetite and glass from decompression experiments of the current study. **(A)** shows an overview BSE image including a typical inclusion-rich (black spots) magnetite core and pristine magnetite rim discovered at Los Colorados. **(B)** is the enlargement of an inclusion in the magnetite core and exhibits its polycrystalline nature. The inclusion-rich magnetite cores observed at Los Colorados are interpreted as igneous magnetite, since polycrystalline silicate inclusions only homogenized at magmatic temperatures (T>975 °C)[43]. The experiments of this study (**C**, **D** and **E**) reveal that sudden supersaturation of the melt caused by decompression/degassing results in fast magnetite growth, such as hopper growth[28], where several silicate melt inclusions can be entrapped within euhedral appearing crystals. The size and habitus of the experimental magnetite inclusions are very similar to those in natural samples. This provides further evidence that polycrystalline silicate inclusions in oxides are an igneous growth feature; i.e., magnetite entraps melt as melt inclusions that crystallize during cooling into polycrystalline silicate inclusions.

Table S1: Electron probe microanalyses (EPMA) data of experimental glasses.

sample #	P1D	09-H_2O	09-Cl	16-H_2O	14-Cl	28-H_2O	28-Cl	01-H_2O	01-Cl
decompression	no	no	no	yes	yes	yes	yes	yes	yes
annealing	-	-	-	0 h	0 h	3 h	3 h	72 h	72 h
Na_2O	3.75	3.81	3.61	3.75	3.59	3.62	3.65	3.60	3.54
K_2O	1.09	1.14	1.13	1.13	1.13	1.06	1.08	1.05	1.08
MgO	1.93	2.02	1.99	1.99	1.97	2.00	2.01	1.99	1.96
Al_2O_3	17.49	18.33	18.29	18.26	18.17	18.54	18.52	18.48	18.52
SiO_2	62.91	63.92	63.77	64.49	64.00	64.62	64.31	64.69	64.21
CaO	6.16	6.31	6.26	6.38	6.22	6.49	6.43	6.45	6.40
TiO_2	0.46	0.36	0.36	0.35	0.36	0.38	0.33	0.36	0.35
FeO	5.59	4.11	4.60	3.65	4.57	3.28	3.69	3.39	3.93
Cl	0.00	0.00	1.20	0.00	1.19	0.00	1.22	0.00	1.03
wt% mgt*	0.00	1.59	1.64	2.08	1.67	2.48	2.62	2.36	2.37

Elemental compositions are normalized to 100 %. P1D represents the composition of the starting glass. *wt% mgt was calculated by difference to the starting composition. For Cl-bearing experiments the addition of Fe induced by the added fluid as $FeCl_3$ (0.54 wt% Fe addition to the system) was taken into account prior to by-difference calculations.

Table S2: FTIR-spectroscopy data of experimental glasses

sample #	09-H_2O	09-Cl	16-H_2O	14-Cl	28-H_2O	28-Cl	01-H_2O	01-Cl
decompression	no	no	yes	yes	yes	yes	yes	yes
annealing	-	-	0 h	0 h	3 h	3 h	72 h	72 h
H_2O total (wt%)	5.96	5.74	5.24	4.94	5.30	5.32	5.14	4.84
StDev (wt%)	0.07	0.13	0.22	0.12	0.06	0.12	0.08	0.18
Δ wt%	-	-	-0.72	-0.80	-0.66	-0.42	-0.82	-0.90

H_2O concentrations were averaged over five measurements across each sample. Δwt% represents the amount of degassed H_2O relative to undegassed samples at 250 MPa (09-H_2O and 09-Cl).

Additional references

[40]Ghiorso, Mark S., and Sack, Richard O. (1995) Chemical Mass Transfer in Magmatic Processes. IV. A Revised and Internally Consistent Thermodynamic Model for the Interpolation and Extrapolation of Liquid-Solid Equilibria in Magmatic Systems at Elevated Temperatures and Pressures. Contributions to Mineralogy and Petrology, 119, 197-212

[41]Asimow PD, Ghiorso MS (1998) Algorithmic Modifications Extending MELTS to Calculate Subsolidus Phase Relations. American Mineralogist, 83, 1127-1131

[42]Giordano, D., Russell, J. K., & Dingwell, D. B. Viscosity of magmatic liquids: a model. *Earth. Planet. Sci. Lett.*, 271(1-4), 123-134 (2008).

[43]Knipping, J.L. et al. Trace elements in magnetite from massive iron oxide-apatite deposits indicate a combined formation by igneous and magmatic-hydrothermal processes. *Geochim. Cosmochim. Acta*, 171, 15-38 (2015b).
